# “Own doctor” presence in a web-based lifestyle intervention for adults with obesity and hypertension: A randomized controlled trial

**DOI:** 10.3389/fpubh.2023.1115711

**Published:** 2023-03-14

**Authors:** Pedro Múzquiz-Barberá, Marta Ruiz-Cortés, Rocío Herrero, María Dolores Vara, Tamara Escrivá-Martínez, Rosa María Baños, Enrique Rodilla, Juan Francisco Lisón

**Affiliations:** ^1^Department of Nursing and Physiotherapy, Faculty of Health Sciences, University CEU-Cardenal Herrera, CEU Universities, Valencia, Spain; ^2^Department of Biomedical Sciences, Faculty of Health Sciences, University CEU-Cardenal Herrera, CEU Universities, Valencia, Spain; ^3^Department of Psychology and Sociology, Universidad de Zaragoza, Teruel, Spain; ^4^CIBER-Obn Physiopathology of Obesity and Nutrition, Instituto de Salud Carlos III, Madrid, Spain; ^5^Universidad Europea de Valencia, Valencia, Spain; ^6^Polibienestar Research Institute, Universitat de València, Valencia, Spain; ^7^Department of Medicine and Surgery, Faculty of Health Sciences, University CEU-Cardenal Herrera, CEU Universities, Valencia, Spain; ^8^Hypertension and Vascular Risk Unit, Hospital Universitario de Sagunto, Valencia, Spain

**Keywords:** e-Health, web-based, multimedia, healthy lifestyle, physical activity, nutrition, obesity, hypertension

## Abstract

**Introduction:**

Online interventions have long been shown to be an effective means to promote a healthy lifestyle, thereby helping to control body weight and blood pressure figures. Likewise, using video modeling is also considered an effective way to guide patients through behavioral interventions. Nonetheless, to the best of our knowledge, this study is the first to analyze how the presence of patients' “own doctor” in the audiovisual content of a web-based lifestyle program (“*Living Better”*) aimed at promoting regular physical exercise and healthy eating behavior, compared with an “unknown doctor,” influences the outcomes of adults with obesity and hypertension.

**Materials and methods:**

A total of 132 patients were randomly assigned either to the experimental (*n* = 70) or control (*n* = 62) group (“own doctor” or “unknown doctor”, respectively). The body mass index, systolic and diastolic blood pressure, number of antihypertensive drugs used, physical activity level, and quality of life was assessed and compared at baseline and post-intervention (12 weeks).

**Results:**

The intention-to-treat analysis showed intragroup significant improvements in both groups in terms of the body mass index (control group: mean difference −0.3, 95% CI [−0.5, −0.1], *p* = 0.002; experimental group: −0.4 [−0.6, −0.2], *p* < 0.001) and systolic blood pressure (control group: −2.3 [−4.4, −0.2], *p* = 0.029; experimental group: −3.6 [−5.5, −1.6], *p*< 0.001). In addition, there were also significant improvements in the experimental group for the diastolic blood pressure (−2.5 [−3.7, −1.2], *p* < 0.001), physical activity (479 [9, 949], *p* = 0.046), and quality of life (5.2 [2.3, 8.2], *p* = 0.001). However, when comparing the experimental with the control group, no between-group significant differences were found in these variables.

**Conclusions:**

This study suggests that the presence of patients' “own doctor” in the audiovisual content of a web-based intervention, aimed at promoting a healthy lifestyle among adults with obesity and hypertension, do not show significant additional benefits over the efficacy of e–counseling.

**Trial registration:**

ClinicalTrials.gov NCT04426877. First Posted: 11/06/2020. https://clinicaltrials.gov/ct2/show/NCT04426877.

## 1. Introduction

The international guidelines specialized in hypertension (1, 2) and obesity (3) agree that the first step to consider in clinical approaches to patients with obesity and hypertension should be the promotion and acquisition of a healthier lifestyle, based on two key pillars: the establishment of healthy eating behavior and regular engagement in physical exercise. On the other hand, with the aim of promoting proactive disease control by patients and reducing the burden of care, the World Health Organization (4) has been trying to encourage health interventions administered through the internet and technologies for many years now. Accordingly, multiple publications have shown the effectiveness of educational interventions leveraging multimedia material in different pathological populations (5–11), with most of them also being oriented toward education about healthy lifestyles (5–7, 11).

Furthermore, “using video modeling, which involves the demonstration of desired behaviors, outcomes, and attitudes through active, visual representations by an actor,” is considered an effective way to educate and guide patients through behavioral interventions, even for people with low levels of literacy (8, 12). Moreover, it has also been shown that the simple gesture of doctors talking to patients about their own personal practices —in terms of physical activity and nutrition— helps promote general patient health. This is because patients are more likely to adopt healthy behaviors when their doctor also practices them (referred to as the “lead by example” practice) (13). Indeed, the therapeutic alliance, understood as the quality of the relationship between the patient and the doctor, seems to be a decisive factor in patients assuming more proactive roles in their own health care (14, 15).

Considering all the above, the objective of this present study was to analyze the influence exerted by the identity of the main doctor appearing in the audiovisual content of our e-Health intervention on patients with the obesity–hypertension phenotype in terms of the following variables: body mass index, systolic and diastolic blood pressure, number of antihypertensive drugs used, physical activity, quality of life, satisfaction and adherence to the intervention. To do this, after 12 weeks of intervention with our “*Living Better”* web-based program (16–19), we compared the results in two groups: the control group in which an “unknown doctor” appeared in the audiovisual content, and the experimental group whose audiovisual content instructions were provided by the patients' own hypertension specialist. We hypothesized that (1) all the participants would achieve improvements in the different variables analyzed after the 12-week intervention, regardless of the identity of the doctor present in the audiovisual content delivered to them and (2), patients who saw their own specialist doctor giving them the indications would attain greater benefits than those in the control group.

## 2. Materials and methods

### 2.1. Study design

This was a prospective, single-center, clinical trial (registered at ClinicalTrials.gov: NCT04426877) with balanced randomization (1:1). This study was reviewed and approved by the University CEU-Cardenal Herrera Ethics Committee (CEI19/085). This research was also approved by the Human Research Ethics Committee at the *Hospital Universitario de Sagunto* and followed the ethical guidelines established in the Declaration of Helsinki.

### 2.2. Eligibility criteria

The following inclusion criteria were applied to select the study participants: adults aged between 18 and 75 years with hypertension who were overweight (body mass index >24.9 kg/m^2^ and <30 kg/m^2^) or who had type I obesity (body mass index >29.9 kg/m^2^ and <35 kg/m^2^), and who were patients that saw the same physician specialized in hypertension. As previously described (18, 19), hypertension was defined as a systolic blood pressure ≥140 mmHg and/or a diastolic blood pressure ≥90 mmHg, or patients taking antihypertensive drugs; in this study all the patients were on antihypertensive treatments. Regarding the exclusion criteria, patients who had not come for at least 1 visit with their specialist in the 5 years prior were excluded from the work. In addition, profiles with previous ischemic heart disease, cerebrovascular disease, serious psychiatric disorders, taking more than 3 antihypertensive medications, with physical impairments that could make it difficult to practice exercise, participating in other treatments for weight loss, who had previously participated in our “*Living Better”* intervention (17–19), and/or without internet access were also excluded from this current study.

### 2.3. Procedure

This study took place in the Hypertension and Vascular Risk Unit at the *Hospital Universitario de Sagunto (Valencia, Spain)* between January and June 2021. All the participants formalized their enrollment by signing their informed consent to participate in the study.

Before the start of the trial, an independent researcher unaware of the study characteristics generated a random sequence using a computerized random number generator; this was concealed from all the other study investigators throughout the entire study period. Randomization was performed with stratification for age, sex, and number of specialist visits. Upon enrollment in the study and after completing the primary and secondary outcome measures, the participants (*N* = 132) were randomly assigned either to the control (*n* = 62) or the experimental group (*n* = 70). It was impossible to mask the group allocation to the participants; however, the outcome evaluators and data analysts were blinded to the treatment allocations.

**Figure 1:**
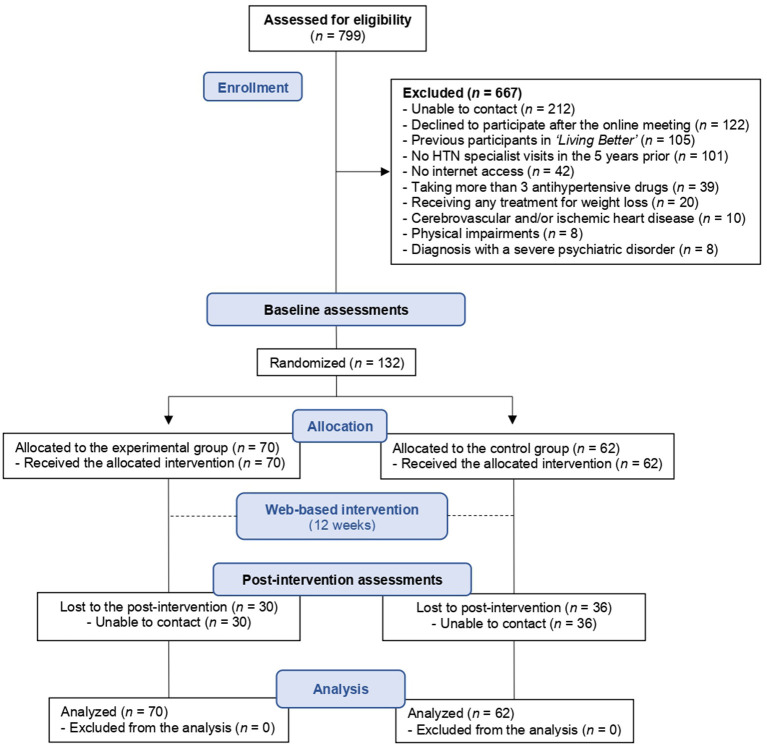
Progression of the participants through the trial.

As shown in the participant flowchart (Figure 1), the different study variables were recorded at baseline just before the start of the program. Once this evaluation was completed, all participants started the 12-week online intervention with the “*Living Better”* web-based program in the group to which they had been previously assigned. The program content followed by both groups was identical, with the exception that the doctor who appeared in the audiovisual material differed between them; the control group patients saw a doctor that they did not know while those assigned to the experimental group saw their own hypertension specialist. Both the doctors involved in delivering the audiovisual content in this study were specialists in hypertension and vascular risk, regularly engaged in physical exercise, and had a healthy appearance. To understand the impact of this intervention on the health of the participants, we recorded all the variables again in the post-intervention assessments at the end of the program.

### 2.4. Intervention

The “*Living Better”* program is a computerized intervention that is self-administered through the internet. The treatment protocol consists of 9 modules and incorporates psychological strategies that encourage a healthy lifestyle by promoting the regular practice of physical exercise and healthy eating behavior. A period of 12 weeks is allowed for completion of the entire program, during which time the modules are activated weekly or fortnightly. Some of the techniques used that have already been described in the literature (20–22) were self-monitoring, self-instruction, behavioral recording, stimulus control, self-reinforcement, problem-solving techniques, and homework. More details about the original intervention can be found in Baños et al. (16), Mensorio et al. (17), and Lison et al. (18). Furthermore, considering the suggestions of the participants in these previous studies (mainly to help facilitate usability) and in order to test our current hypothesis, we converted part of the written content into audiovisual materials, as detailed in Múzquiz-Barberá et al. (19). As previously mentioned, the content was identical in both groups, but the doctor who appeared in the audiovisual material differed between the groups. The audiovisual presence of the doctors (presented in video format) consisted of welcoming the patients and establishing the objectives of the module, demonstrating the exercises the participants had to practice, concluding the module and introducing the next one, and encouraging the participants to continue advancing through the intervention and put everything they had learned into practice. Specifically, “*Living Better”* contains 32 videos that total 52 min of the presenting doctors' audiovisual presence.

### 2.5. Outcome measures

Patient age, sex, time since the hypertension diagnosis, and the number of visits to the specialist since the first diagnosis as hypertensive, were all registered before the randomization process was implemented. Furthermore, the variables listed below were recorded before and after the intervention, through the same platform as the intervention program.

#### 2.5.1. Primary outcome

##### 2.5.1.1. Body mass index

Due to the indications of the health authorities and the hospital regulations related to the COVID-19 pandemic, the participants were instructed to register this variable in a pharmacy near their home. They were also instructed to avoid smoking for 48 h, caffeine for 12 h, and strenuous exercise for 24 h prior to the registration. In addition, they were asked to go the pharmacy while fasting to avoid the possibility that any food or drink ingested could influence their data. Thus, the same person (pharmacist or pharmacy assistant) used an approved device to assess the different body composition variables for each patient. Body mass index was calculated by dividing patient weight by their height squared (kg/m^2^).

#### 2.5.2. Secondary outcomes

##### 2.5.2.1. Systolic and diastolic blood pressure

The patient's body composition measurements and systolic and diastolic blood pressure were also recorded at the same pharmacy. This was done first thing in the morning and before taking their antihypertensive medication to avoid possible alterations in the measurements. Blood pressure was strictly analyzed according to the American College of Cardiology/American Society of Hypertension (1) and the European Society of Hypertension (ESH)/European Society of Cardiology guidelines (2). Of note, the participants of this study, and in general every patient treated in the Hypertension Unit at the *Hospital Universitario de Sagunto*, are routinely trained to correctly measure blood pressure in this way.

##### Number of antihypertensive drugs

The patients recorded the number of prescribed medications they used to control of their hypertension.

##### Physical activity level

The short version of the *International Physical Activity Questionnaire* (IPAQ-SF) was used (23, 24) to assess the time each subject had spent being active in the 7 days prior to completion of the survey.

##### Quality of life

The *SF-12 Health Questionnaire* (a reduced version of the SF-36) was applied to measure quality of life (25). This self-administered instrument provides a health status profile and consists of 12 items grouped into the 8 dimensions of the SF-36, ranging from 0 (the worst state of health for that dimension) to 100 (the best state of health) (25). In the current study, we focused on the analysis of the General Health dimension.

At the end of the intervention, we recorded the adherence of the participants to the program. To do this, we took advantage of the data regarding the degree of completion of each patient collected automatically by the online platform. In other words, we recorded how many modules they had reviewed out of a total of 9, and how much time they had spent on average per module. This also made it possible to estimate the minutes of audiovisual content (in video format) that each participant had viewed. Finally, the participants registered their degree of general satisfaction with the intervention on a scale from 0 (least satisfaction) to 10 (maximum satisfaction).

### 2.6. Statistical analysis

To detect a reduction in body mass index of 1 ± 1.7, which agrees with the data of a previous study (17), with a two-sided 5% significance level and a power of 80%, and also accounting for an anticipated dropout rate of 30%, a sample size of 60 participants per group was required. The statistical analysis was performed according to intention-to-treat. We used SPSS software for Windows (version 19.0; IBM Corp., Armonk, NY) in all our analyses.

Two-way mixed analysis of covariance (ANCOVA) tests was used to compare the study effects on body mass index, systolic and diastolic blood pressure, physical activity, and quality of life, using time as the within-group factor (baseline vs. post-intervention assessments), and the group as the between-group factor (control vs. experimental group). The analysis was adjusted for number of antihypertensive drugs. On the other hand, we implemented a two-way mixed ANOVA test for the antihypertensive drugs variable, also using time as the within-group factor (baseline vs. post-intervention assessments) and the group as the between-group factor (control vs. experimental group).

Bonferroni *post-hoc* tests were applied following the ANCOVAs and ANOVA. Partial eta-squared (ηp^2^) effect sizes were calculated such that 0.01–0.06, 0.06–0.14, and 0.14 or higher, respectively corresponded to small, medium, and large effect sizes (26). Non-parametric Mann–Whitney U tests were used to calculate the degree of satisfaction and adherence to the intervention (number of modules completed and time spent per module) by participants assigned to the control and experimental group, respectively. A per-protocol analysis was performed to compare the study effects which would occur under optimal conditions. Finally, correlation analyses were performed to examine possible associations between the changes (post-intervention minus the baseline) in body mass index, systolic and diastolic blood pressure, physical activity level, number of antihypertensive drugs, and quality of life. The magnitude of the Pearson correlation was interpreted according to the suggestions by Hopkins et al. (27) where 0.0–0.1 = trivial; 0.1–0.3 = small; 0.3–0.5 = moderate; 0.5–0.7 = large; 0.7–0.9 = very large, and 0.9–1 = almost perfect. In addition, forward stepwise regression was used to determine the combination of variables that most accurately predicted quality of life of patients. The statistical significance was set at *p* < 0.05 for all our analyses.

## 3. Results

### 3.1. Reported changes on the body mass index, blood pressure, antihypertensive drugs, physical activity, and quality of life

Table 1 shows the values collected during the baseline assessment, prior to the intervention. Table 2 shows the results of the tests according to an intention-to-treat analysis. As shown, the two-way mixed ANCOVA tests showed intragroup significant improvements in body mass index and systolic blood pressure in the control group with a moderate and small effect size, respectively. There were intragroup significant improvements in all the variables analyzed in the patients assigned to the experimental group, with moderate effect sizes for body mass index, systolic and diastolic blood pressure, and quality of life. As shown in Supplementary Table 1, the per-protocol analysis executed showed significant improvements with a large effect size for body mass index in both groups, and also in the experimental group for systolic and diastolic blood pressure, and quality of life. The two-way mixed ANOVA test results did not show any intragroup significant changes in the number of antihypertensive drugs used by the participants in either group. However, when comparing the experimental with the control group after the intervention, no between-group differences were found in any analysis (Table 3 and Supplementary Table 2), except for antihypertensive drugs variable in which statistical significance were found in both intention-to-treat (−0.3 [−0.6, −0.1]; *p* = 0.011) and per-protocol (−0.5 [−0.9, −0.1]; *p* = 0.008) analysis; this difference had already been found before the intervention (*p* = 0.012). Associations between the changes (post-intervention minus the baseline) in body mass index, systolic and diastolic blood pressure, physical activity level, number of antihypertensive drugs, and quality of life are summarized in Supplementary Table 3. Stepwise multiple regression revealed that the changes in physical activity level was a significant and independent predictor for the improvement in quality of life (AdjR2 = 0.092, β = 0.315, *p* < 0.001; model 1), explaining 9.2% of the variation in the quality of life (Supplementary Table 4). Model 2 included the systolic blood pressure to the physical activity level and explained 14.8 % of the variation.

**Table 1:** Baseline characteristics of the participants.

**Variables**		**Control group (*n* = 62), mean (*SD*)^a^**	**Experimental group (*n* = 70), mean (*SD*)^a^**
Sex (*n*)	Women	28	32
	Men	34	38
Age (years)		57.7 (10.7)	56.2 (9.5)
Hypertension diagnosis (years)		10.3 (8.2)	10.4 (9.2)
Specialist visits (*n*)		9.8 (8.3)	9.4 (7.9)
Weight (kg)		84.6 (13.7)	82.3 (12.4)
Body mass index (kg/m^2^)		29.6 (3.2)	29.6 (3.7)
Systolic blood pressure (mmHg)		128.8 (11.3)	131.8 (12.6)
Diastolic blood pressure (mmHg)		80.5 (8.3)	81.8 (9.1)
Antihypertensive drugs (*n*)		1.4 (0.9)	1.1 (0.6)
Physical activity level (METs-min/week)		2731 (3326)	3013 (3270)
Quality of life (points)		46.9 (15.1)	40.1 (22.8)

a Average values previous to patients' intervention.

**Table 2:** Intention-to-treat analysis. Intragroup comparisons: baseline vs. post-intervention.

	**Control group (*****n*** = **62)**			**Experimental group (*****n*** = **70)**			**ANOVA effects (** ***p*** **-value)**		
**Variables**	**Difference (95% CI)** ^a^	**Partial eta** ^2^	***p*** **-value**	**Difference (95% CI)** ^a^	**Partial eta** ^2^	***p*** **-value**	**Time**	**Group**	**Time** × **group**
Body mass index (kg/m^2^)	−0.3 (−0.5, −0.1)	0.073	0.002^**^	−0.4 (−0.6, −0.2)	0.133	<0.001^**^	0.001^**^	0.911	0.469
Systolic blood pressure (mmHg)	−2.3 (−4.4, −0.2)	0.037	0.029^*^	−3.6 (−5.5, −1.6)	0.095	<0.001^**^	0.001^**^	0.261	0.378
Diastolic blood pressure (mmHg)	−0.9 (−2.3, 0.4)	0.015	0.162	−2.5 (−3.7, −1.2)	0.108	< 0.001^**^	0.003^**^	0.737	0.102
Antihypertensive drugs (*n*)	−0.1 (−0.2, 0.0)	0.019	0.115	−0.1 (−0.2, 0.0)	0.017	0.136	0.031^*^	0.009^**^	0.906
Physical activity level (METs-min/week)	175 (−318, 669)	0.004	0.483	479 (9, 949)	0.033	0.046^*^	0.598	0.447	0.384
Quality of life (points)	1.9 (−1.2, 5.0)	0.012	0.228	5.2 (2.3, 8.2)	0.095	0.001^**^	0.528	0.133	0.125

a Difference was calculated as the post-intervention minus the baseline.

* *p* ≤ 0.05;

** *p* ≤ 0.01.

**Table 3:** Intention-to-treat analysis. Between-group comparisons: control vs. experimental group.

	**Baseline**					**Post-intervention**				
**Variables**	**Control Group (*****n*** = **62)**	**Experimental Group (*****n*** = **70)**	**Difference (95% CI)** ^a^	**Partial eta** ^2^	***p*** **-value**	**Control Group (*****n*** = **62)**	**Experimental Group (*****n*** = **70)**	**Difference (95% CI)** ^a^	**Partial eta** ^2^	***p*** **-value**
Body mass index (kg/m^2^)	29.6 (3.2)	29.6 (3.7)	0.0 (−1.3, 1.2)	0.000	0.972	29.3 (3.5)	29.2 (3.7)	−0.1 (−1.4, 1.2)	0.000	0.852
Systolic blood pressure (mmHg)	128.8 (11.3)	131.8 (12.6)	3.0 (−1.3, 7.2)	0.015	0.173	126.5 (11.9)	128.2 (12.6)	1.7 (−2.7, 6.0)	0.005	0.445
Diastolic blood pressure (mmHg)	80.5 (8.3)	81.8 (9.1)	1.3 (−1.9, 4.4)	0.005	0.428	79.6 (8.0)	79.3 (8.4)	−0.3 (−3.2, 2.6)	0.000	0.852
Antihypertensive drugs (*n*)	1.4 (0.9)	1.1 (0.6)	−0.3 (−0.6, −0.1)	0.049	0.012^*^	1.3 (0.8)	1.0 (0.6)	−0.3 (−0.6, −0.1)	0.050	0.011^*^
Physical activity level (METs-min/week)	2731 (3326)	3013 (3270)	282 (−921, 1485)	0.002	0.643	2907 (3073)	3492 (3220)	586 (−564, 1735)	0.008	0.315
Quality of life (points)	46.9 (15.1)	40.1 (22.8)	−6.8 (−13.9, 0.3)	0.029	0.061	48.8 (15.8)	45.3 (21.6)	−3.5 (−10.4, 3.5)	0.029	0.328

a Difference was calculated as the experimental minus the control group.

* *p* ≤ 0.05;

^**^p ≤ 0.01.

### 3.2. Differences showed on adherence and satisfaction with the intervention

Regarding adherence to the intervention, no statistically significant differences were observed between the control and the experimental group in terms of the median number of modules completed (control group: 4.0 [IQR = 5]; experimental group: 4.5 [IQR = 5]; *p* = 0.982) or the median minutes dedicated to each module (control group: 60.0 [IQR = 25]; experimental group: 60.0 [IQR = 35]; *p* = 0.710). Specifically, 42 and 49% of the participants in the control and experimental group reached the middle of the program (5 modules or more), respectively. Finally, both groups showed similar median levels of patient satisfaction with the intervention and there were no statistically significant differences between them (control group: 8.0 [IQR = 4]; experimental group: 8.0 [IQR = 4]; *p* = 0.621).

## 4. Discussion

To the best of our knowledge, this is the first study to analyze the influence of the audiovisual presence of the patients' own specialist doctor in an online intervention program aimed at promoting a healthy lifestyle (regular physical exercise and healthy eating behavior) in patients with an obesity–hypertension phenotype. Indeed, we are not aware of any other studies on any other disease or pathological condition that have looked at this possible effect. Contrary to expectation, the presence of a patients' “own doctor” in the audiovisual content did not bring about any significant additional benefits over the efficacy of the e–counseling in patients with obesity and hypertension.

It is known that physically active and health-conscious doctors can become influential role models for their patients, motivating them to adopt healthier lifestyles with the aim of preventing and treating possible chronic diseases (13, 28). In fact, health promotion by physicians is more effective than outsourcing advice to a health coach, in part because patients view physicians as the most authoritative source in which to entrust their health (13). Furthermore, several lines of investigation (29–31) have found that the therapeutic alliance can be just as effective when treatments are carried out online or making use of technological platforms. With the aim of improving the therapeutic alliance and, therefore, the results of our primary and secondary outcomes, a total of 32 videos were included in the intervention, resulting in a combined 52 min of audiovisual contact with the presenting doctor (~6 min per module). However, despite the significant benefits reported by both groups in the intragroup analysis, the possible influence exerted by the identity of the main doctor appearing in the audiovisual content did not reach the statistical significance in the between-group comparisons.

The degree of completion by the participants was lower compared to a previous “*Living Better”* study (18), corresponding to 24% less adherence to the intervention. This may be the result of participant difficulties in the context of the ongoing COVID-19 pandemic or in following the demanding planning presented by the program in relation to accessing and reviewing the different modules. Likewise, the number of losses in the post-intervention assessments was also manifestly higher (43% in the experimental and 58% in the control group) compared to those (18%) in the previous study (18). This may have been due to an increase in the difficulty in taking the post-intervention measurements which had to be carried out outside of the hospital context because of the health restrictions due to COVID-19. Despite these two differences (lower adherence and increased losses after the intervention), the results of the intention-to-treat statistical analysis showed that all participants experienced benefits. Furthermore, in order to verify if the low adherence and the high losses conditioned the possible effect of the presence of their “own doctor,” it was decided to carry out a per-protocol analysis to analyze the real impact of “*Living Better”* on the participants who had completed at least 5 modules and had undertaken the post-intervention assessments. Interestingly, in line with our intention-to-treat analysis, the per-protocol analysis did not show between-group differences after the intervention, confirming the observed result that the inclusion of a patients' “own doctor” did not result in significant additional benefits.

Although the results of this study did not confirm the initial hypothesis, the “*Living Better”* program has again shown to have benefits on the different study variables. In this sense, the results suggest that an intervention of this nature can improve body composition, blood pressure, levels of physical activity and quality of life in patients with obesity and hypertension. The results showed positive correlations between the improvements in body mass index and systolic and diastolic blood pressure. In this sense, the academic literature reflects the direct impact that weight reduction, and therefore body mass index, has on blood pressure values (32, 33). Indeed, a meta-analysis (33) showed that a decrease in blood pressure figures of approximately 1 mmHg is achieved for each kilogram lost. In addition, systolic blood pressure reductions of 5 mmHg have been associated with significant reductions in all-cause mortality (34). At this point, we must remember that educational interventions with multimedia materials are considered potentially more effective than other forms of support when trying to address physical inactivity and obesity (35). Likewise, the provision of online advice through videos also facilitates the learning of new behavior related to health (7, 36). Thus, this type of intervention has widely demonstrated its effectiveness in controlling body weight (37–44) and blood pressure figures (45–48) by promoting a healthy lifestyle. In fact, the participants in the experimental group showed greater benefits than those participants in the previous studies implementing the original “*Living Better”* intervention (17–19). Perhaps, these differences could be explained by the addition of more multimedia content in this current version of “*Living Better*.”

### 4.1. Limitations

It is important to outline the limitations of this study. Firstly, the enrolled participants had demonstrated an initial level of motivation to engage in an e-Health program, which may have introduced potential selection bias. Secondly, this single-center clinical trial only involved one doctor per arm, and therefore is potentially confounded by their personal characteristics that could have influenced the outcomes. In addition, we did not control the analysis for confounding psychological variables such as therapeutic alliance or similar constructs. A third possible limitation includes recall bias, because all the participants' responses in the questionnaires were conditioned by their ability to recall their habits, as well as desirability bias, whereby participants tended to minimize unhealthy habits and exaggerate healthy behaviors. Also, there was a low adherence to the intervention and a high attrition rate at the post-intervention assessments, so that could perhaps have limited the between-group differences. Finally, information about the results in terms of the systolic and diastolic blood pressure achieved were limited; firstly, because the data were self-reported and could not be verified (because of the COVID-19 pandemic restrictions), and secondly, because ambulatory blood pressure measurement would have been a more accurate method to assess changes in blood pressure values.

## 5. Conclusions

This study suggests, for the first time, that the presence of patients' “own doctor” in the audiovisual content of an online intervention program (aimed at promoting a healthy lifestyle through regular physical exercise and healthy eating behavior) do not show significant additional benefits over the efficacy of the e–counseling in patients with an obesity–hypertension phenotype. Future studies with multiple doctors per arm, controlling for therapeutic alliance or similar constructs, with a larger and more representative sample size, and with ambulatory blood pressure measurements, should investigate the impact of the presence of a patients' “own doctor” in audiovisual web-based interventions for adults with obesity and hypertension. On the other hand, this study opens the door to future research on online interventions focused on other pathological populations supported by multimedia material and the presence of a physician or other health professionals.

## Data availability statement

The raw data supporting the conclusions of this article will be made available by the authors, without undue reservation.

## Ethics statement

The studies involving human participants were reviewed and approved by University CEU-Cardenal Herrera Ethics Committee (CEI19/085). The patients/participants provided their written informed consent to participate in this study.

## Author contributions

PM-B, ER, RB, and JL conceived this research methodology and wrote/prepared the original draft. MR-C and RH were responsible for the methodology. PM-B and JL conducted a formal analysis. RB, ER, and JL managed the investigation. PM-B, MR-C, and RH reviewed and edited the manuscript. MV and TE-M were responsible for visualization. All authors contributed to the article and approved the submitted version.
