# Supplementary material for: “Own doctor” presence in a web-based lifestyle intervention for adults with obesity and hypertension: A randomized controlled trial
Source: Front Public Health. 2023 Mar 14;11:1115711. doi: 10.3389/fpubh.2023.1115711 (PMC10043391; doi:10.3389/fpubh.2023.1115711)
Supplement: Supplementary file 1 [file Table_1.pdf]

TABLE S1. Per-protocol analysis. Intragroup comparisons: baseline versus post-intervention.

	**Control Group** (*n*=26)			**Experimental Group** (*n*=40)			**ANOVA effects** (*p* value)		
**VARIABLES**	Difference (95% CI) [a]	Partial eta^2	*p* value	Difference (95% CI) [a]	Partial eta^2	*p* value	Time	Group	Time × Group
**Body mass index** (kg/m^2)	−0.9 (−1.3, −0.5)	.288	<.001**	−1.1 (−1.5, −0.7)	.407	<.001**	<.001**	.065	.578
**Systolic blood pressure** (mmHg)	−7.1 (−12.3, −1.9)	.149	.009**	−9.4 (−14.1, −4.6)	.269	<.001**	<.001**	.237	.529
**Diastolic blood pressure** (mmHg)	−2.9 (−6.3, 0.5)	.064	.094	−6.6 (−9.7, −3.5)	.298	<.001**	.002**	.958	.125
**Antihypertensive drugs** (*n*)	−0.2 (−0.5, 0.0)	.055	.101	−0.2 (−0.4, 0.1)	.044	.144	.030*	.004**	.790
**Physical activity level** (METs-min/week)	432 (−586, 1450)	.011	.399	775 (−53, 1604)	.053	.066	.561	.459	.605
**Quality of life** (points)	4.7 (−1.8, 11.3)	.034	.155	8.8 (3.6, 14.1)	.163	.001**	.405	.121	.336

[a] Difference was calculated as the post-intervention minus the baseline.
*$p \leq 0.05$; **$p \leq 0.01$.
