# Supplementary material for: “Own doctor” presence in a web-based lifestyle intervention for adults with obesity and hypertension: A randomized controlled trial
Source: Front Public Health. 2023 Mar 14;11:1115711. doi: 10.3389/fpubh.2023.1115711 (PMC10043391; doi:10.3389/fpubh.2023.1115711)
Supplement: Supplementary file 2 [file Table_2.pdf]

TABLE S2. Per-protocol analysis. Between-group comparisons: control versus experimental group.

VARIABLES	**Baseline** Control Group (*n*=26)	Experimental Group (*n*=40)	Difference (95% CI) [a]	Partial eta^2	*p* value	**Post-intervention** Control Group (*n*=26)	Experimental Group (*n*=40)	Difference (95% CI) [a]	Partial eta^2	*p* value
**Body mass index** (kg/m^2)	28.5 (2.8)	30.4 (3.2)	1.9 (0.0, 3.9)	.087	.049*	27.5 (3.1)	29.3 (3.3)	1.8 (−0.3, 3.8)	.065	.092
**Systolic blood pressure** (mmHg)	126.9 (8.8)	131.5 (13.8)	4.6 (−2.9, 12.1)	.035	.219	119.7 (8.0)	122.1 (11.4)	2.4 (−3.9, 8.6)	.013	.452
**Diastolic blood pressure** (mmHg)	79.1 (8.4)	81.1 (10.6)	2.0 (−4.2, 8.1)	.010	.522	76.2 (6.5)	74.5 (6.9)	−1.7 (−6.0, 2.6)	.015	.423
**Antihypertensive drugs** (*n*)	1.6 (1.0)	1.0 (0.5)	−0.6 (−1.0, −0.2)	.136	.008**	1.3 (0.8)	0.8 (0.5)	−0.5 (−0.9, −0.1)	.138	.008**
**Physical activity level** (METs-min/week)	3574 (3769)	2843 (3051)	−731 (−2458, 997)	.011	.401	4006 (3057)	3619 (2964)	−387 (−1932, 1158)	.004	.618
**Quality of life** (points)	45.1 (14.7)	35.3 (23.0)	−9.8 (−20.6, 1.0)	.054	.073	49.9 (16.5)	44.2 (22.1)	−5.7 (−16.5, 5.0)	.019	.291

[a] Difference was calculated as the experimental minus the control group.
*$p \leq 0.05$; **$p \leq 0.01$.
