# Supplementary material for: “Own doctor” presence in a web-based lifestyle intervention for adults with obesity and hypertension: A randomized controlled trial
Source: Front Public Health. 2023 Mar 14;11:1115711. doi: 10.3389/fpubh.2023.1115711 (PMC10043391; doi:10.3389/fpubh.2023.1115711)
Supplement: Supplementary file 3 [file Table_3.pdf]

TABLE S3. Pearson correlation coefficients between the changes (post-intervention minus the baseline) in the different variables.

	BMI	SBP	DBP	PAL	AD	QoL
BMI	1	.496**	.273**	.000	.162	-.061
SBP		1	.738**	.029	-.142	-.240**
DBP			1	.146	-.258**	-.053
PAL				1	-.092	.315**
AD					1	-.134
QoL						1

BMI = Body Mass Index; SBP = Systolic Blood Preasure; DBP = Diastolic Blood Preasure; PAL = Physical Activity Level; AD = Antihypertensive Drugs; QoL = Quality of Life. **$p \leq 0.01$.
