# Supplementary material for: “Own doctor” presence in a web-based lifestyle intervention for adults with obesity and hypertension: A randomized controlled trial
Source: Front Public Health. 2023 Mar 14;11:1115711. doi: 10.3389/fpubh.2023.1115711 (PMC10043391; doi:10.3389/fpubh.2023.1115711)
Supplement: Supplementary file 4 [file Table_4.pdf]

TABLE S4. Multiple stepwise linear regression analyses with the Quality of Life as dependent variable.

Independent variables	R^2	Adjusted R^2	R^2 change	Standardized β coefficient	Collinearity statistics	
					Tolerance	VIF
Model 1	.099	.092	.099			
PAL				.315**	1.000	1.000
Model 2	.162	.148	.063			
PAL				.323**	.999	1.001
SBP				-.251**	.999	1.001

PAL = Physical Activity Level; SBP = Systolic Blood Preasure. **$p \leq 0.01$.
