# Supplementary material for: “Own doctor” presence in a web-based lifestyle intervention for adults with obesity and hypertension: A randomized controlled trial
Source: Front Public Health. 2023 Mar 14;11:1115711. doi: 10.3389/fpubh.2023.1115711 (PMC10043391; doi:10.3389/fpubh.2023.1115711)
Supplement: Supplementary file 6 [file Data_Sheet_1.pdf]

‘LIVING BETTER’ PROGRAM

The online program ‘Living Better’ is a computerized and self-applied intervention through the Internet (https://psicologiaytecnologia.labpsitec.es/). A 12-week period is established to complete the entire program, during which the modules are activated weekly or fortnightly. The treatment protocol consists of 9 modules and incorporates psychological strategies that encourage a healthy lifestyle by promoting regular physical exercise and healthy eating behaviors. All of them contain multimedia elements (videos, images, texts, and downloadable files) that are presented in a sequential way to advance step by step. Some of the techniques applied in the program consist of self-management, self-instruction, behavioral recording, stimulus control, self-reinforcement, as well as problem-solving techniques.

As for the structure of each module, first, review questions related to the previous module are posed, the objectives of the module in question are established, the contents are explained, and exercises and self-testing questions are proposed to help understand the content. Subsequently, the tasks and physical exercise that the patient should put into practice during the following days until the activation of the next module are indicated; the type and intensity of the physical exercise changes and increases in parallel with the progress of the program. It should be noted that, while the program is active, the system allows the patient to review the content as many times as necessary.

vivir mejor

The web platform includes two interactive tools that are available during the intervention: 1) the Activity Diary, which allows recording activities related to eating habits and physical activity, and 2) the Feedback Section, which allows visualizing through graphs the progress towards the establishment of new weekly habits. In addition, the program also includes an evaluation protocol that will be activated in the pre-intervention phase, after the completion of each module, in the post-intervention, and in the respective follow-up evaluations.

On the other hand, the online platform is configured to send a reminder email if participants fail to access the modules for more than two weeks. This email informs and encourages them to continue with the program, reminding them of the importance of completing the tasks. After three weeks without access to the intervention, a phone call is made to resolve any difficulties or doubts related to the use of the online protocol.

The following is a brief description of the content of each of the modules of the online program ‘Living Better’.

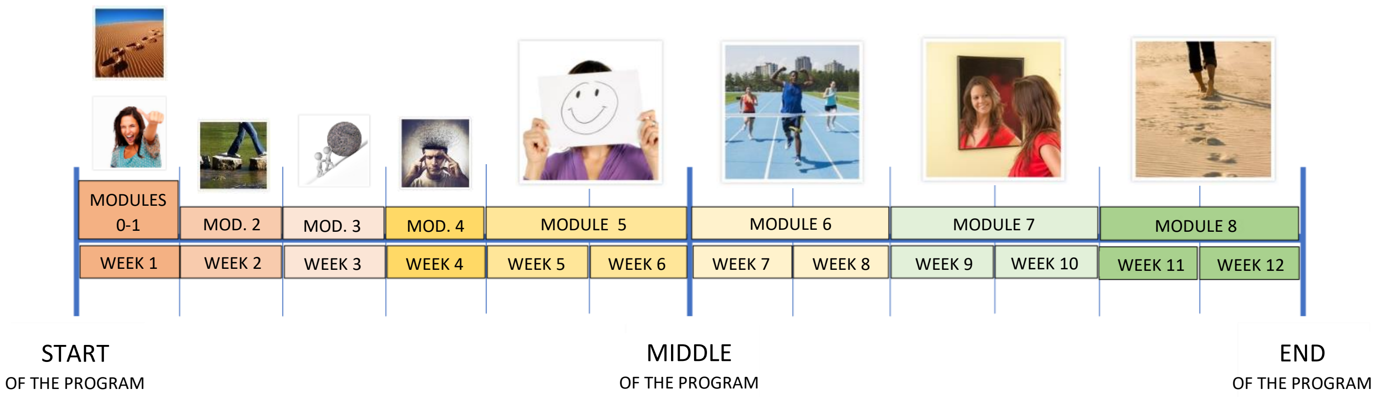

vivir mejor

MODULE 0 – WELCOME

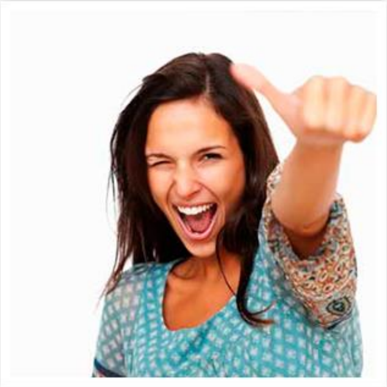

WELCOME

CONTENTS

ACTIVITIES

SELF-TESTING

HOMEWORK

END

The purpose of this module is to welcome participants, describe what the ‘Living Better’ program consists of, detail the structure and distribution of the 9 modules, explain the objectives and main contents, and motivate participants to start the program in order to benefit from it as soon as possible.

vivir mejor

MODULE 1 – GETTING READY TO CHANGE MY LIFESTYLE

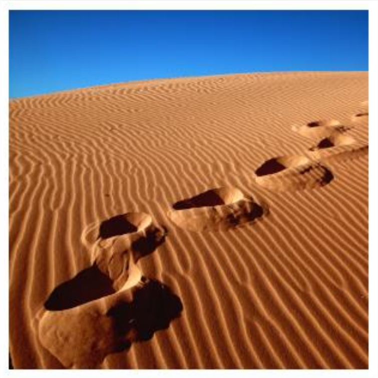

The objective of this module is to provide information on the importance of motivation in lifestyle change. It delves into the costs and benefits of maintaining certain habits and, conversely, the costs and benefits of change. Finally, specific and manageable objectives are established to achieve the proposed changes.

vivir mejor

MODULE 2 – MY WAY TO A HEALTHY LIFESTYLE

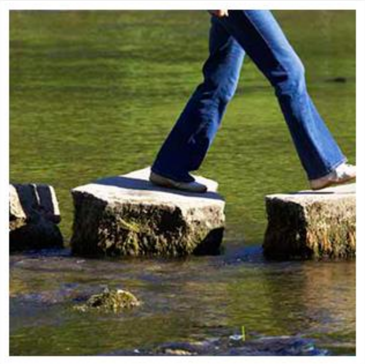

This module reflects on the benefits of a good diet, as well as the role that physical activity plays in our daily lives. Finally, it provides keys to start being more active in our daily lives.

vivir mejor

MODULE 3 – IDENTIFYING BARRIERS

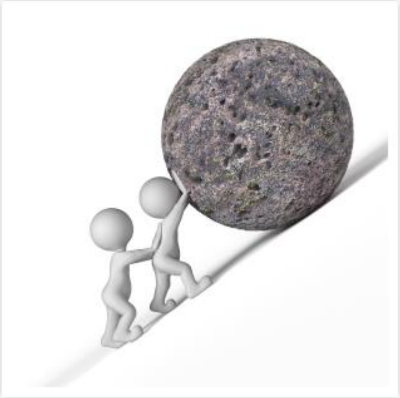

This module tries to provide guidelines to identify the barriers that hinder the adoption of a healthy diet and the regular practice of physical exercise. In addition, different alternatives and/or solutions are proposed to face and overcome them in the best way, offering advice at mealtimes, taking awareness during meals, as well as individualizing and progressing in the dose of physical exercise to avoid excessive fatigue and abandonment.

vivir mejor

MODULE 4 – THE INFLUENCE OF MY THOUGHTS

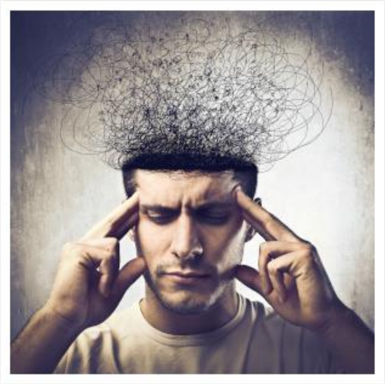

WELCOME
CONTENTS
ACTIVITIES
SELF-TESTING
HOMEWORK
END

The objective of this module is to learn about the role that thoughts play in making choices regarding eating and physical activity habits. Awareness strategies are provided for thoughts that may be interfering with goals or objectives. Specifically, the ABC technique is explained along with tips for healthy eating and a more active lifestyle.

vivir mejor

MODULE 5 – REGULATING MY EMOTIONS

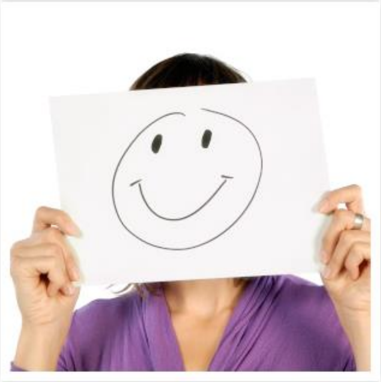

WELCOME

CONTENTS

ACTIVITIES

SELF-TESTING

HOMEWORK

END

This module provides information related to emotional eating. Participants are helped to identify the role that emotion regulation plays in eating behavior. In addition, self-control and mindfulness techniques are provided as alternative strategies to manage emotions.

vivir mejor

MODULE 6 – OVERCOMING MY BARRIERS

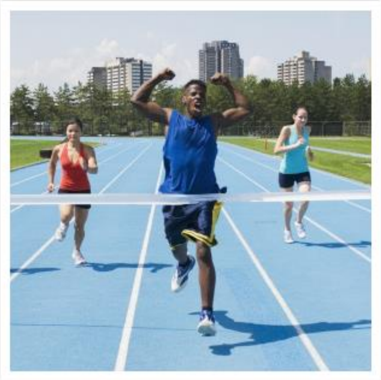

WELCOME

CONTENTS

ACTIVITIES

SELF-TESTING

HOMEWORK

END

The objective of this module is to provide more information about the obstacles and barriers that often appear in the change process, and to teach new coping strategies such as the problem-solving technique.

vivir mejor

MODULE 7 – LOOKING AT ME IN THE MIRROR

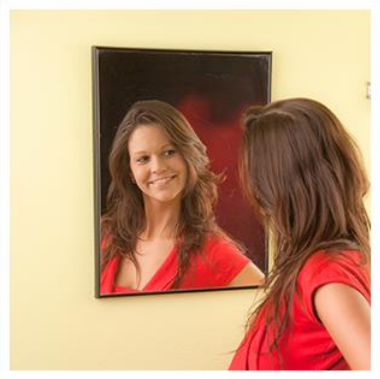

WELCOME

CONTENTS

ACTIVITIES

SELF-TESTING

HOMEWORK

END

This module provides information on the impact of body image on health and well-being. Participants are helped to identify possible concerns related to their body image, and to work on developing a positive body image. Finally, they learn what assertiveness is and some techniques for putting it into practice.

vivir mejor

MODULE 8 – WHAT'S NEXT…?

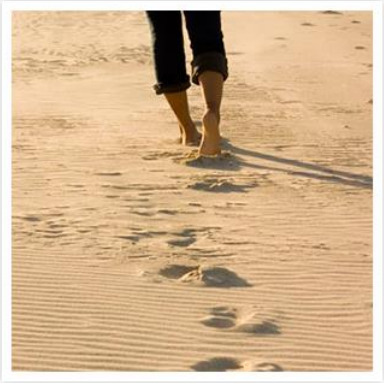

WELCOME

CONTENTS

ACTIVITIES

SELF-TESTING

HOMEWORK

END

The objective of this last module is to summarize all the concepts and techniques shown in the different modules of the 'Living Better' program, reinforcing the changes made, and establishing strategies to maintain the changes obtained and prevent possible relapses.

vivir mejor
