# Supplementary material for: “Own doctor” presence in a web-based lifestyle intervention for adults with obesity and hypertension: A randomized controlled trial
Source: Front Public Health. 2023 Mar 14;11:1115711. doi: 10.3389/fpubh.2023.1115711 (PMC10043391; doi:10.3389/fpubh.2023.1115711)

PHYSICAL EXERCISE PROGRAM

The physical exercise program detailed below is **based on the latest scientific evidence**. In this sense, it aims to **achieve the maximum possible benefits in terms of health improvement**, not only for the treatment of arterial hypertension, but also for the prevention/treatment of many other diseases.

Generally speaking, **a warm-up, aerobic walking exercise, as well as a circuit of simple muscle strengthening, balance and flexibility exercises are included**. As for **the progression in the intensity of the sessions, this has to be adapted to the effort you perceive** when performing each of the activities; as you improve your physical condition, you will feel able to increase the number of repetitions and the demand of the exercises, without increasing your perception of effort.

Remember to quantify the intensity of the session with the methods we have shown you in the program (Speech Test or Borg Scale), in order to better control your level of effort...

Before proceeding, we remind you of the following indications:

- Perform the exercises **during the respiratory exhalation phase**, i.e., while releasing the air. **Always avoid apnea** (stop breathing) when exercising.
- **Do not make sudden changes of position** during exercise to avoid dizziness.
- Do the proposed exercise at least **one hour after taking medication**.
- Do **not** exercise just **before or just after eating**.
- Remember to **control the intensity** at which you exercise (Speech Test, Borg Scale).
- Preferably, do your **physical exercise in the morning**, as you will have more energy.
- **Record the activity** you do on a daily basis to analyze and guide your progress.
- Find a **large space** to perform the exercises comfortably.
- Use **appropriate clothing and materials** for exercise.

<u>WEEKS 1 – 2:</u> Familiarization towards more active habits (and for those who already exercise, to continue to do so).

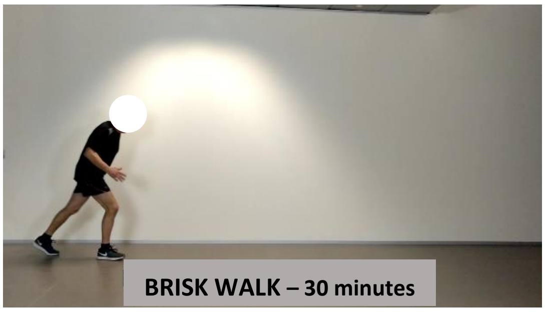

\- \- \- \- \- \- \- \- \- \- \- \-

<u>WEEKS 3 – 4:</u> Brisk walking 2 days per week (30') and the following 4 **exercises 2 days per week (15-30')**.

WARM-UP

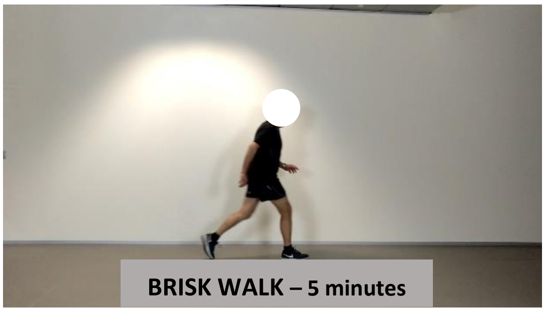

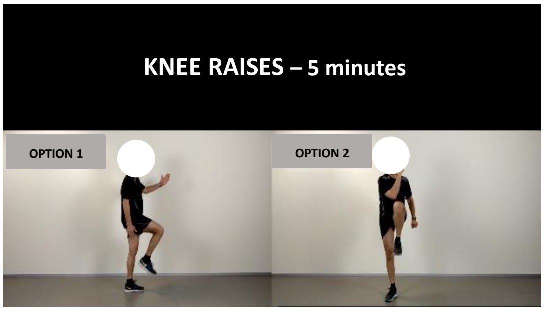

CIRCUIT OF EXERCISES

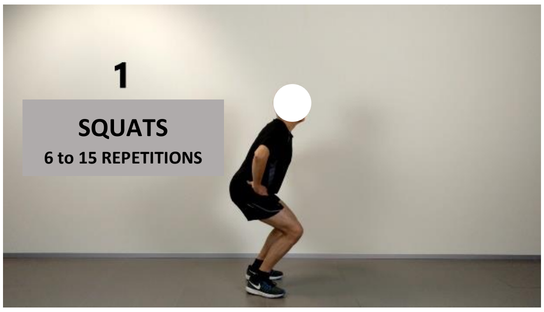

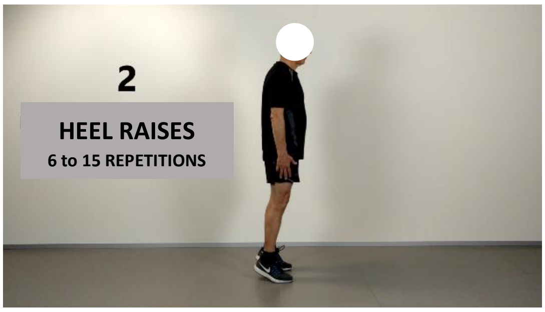

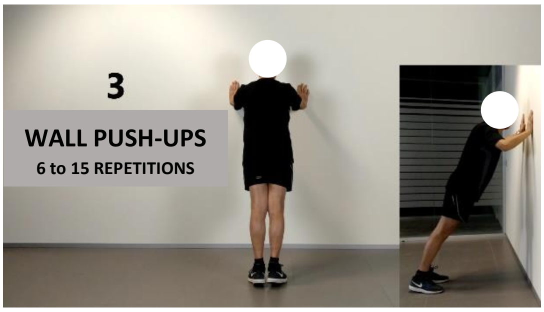

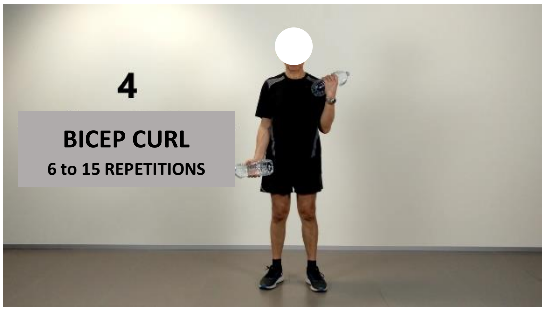

AEROBIC BRISK WALKING EXERCISE

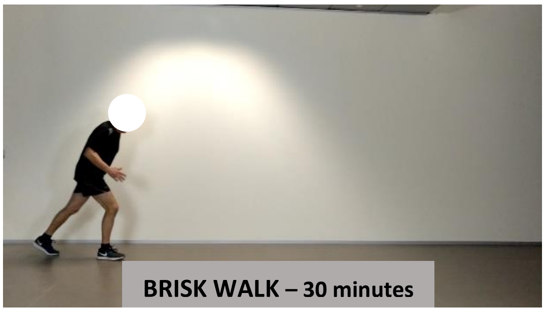

<u>WEEKS 5 – 6:</u> Brisk walking 2 days per week (30') and the following 8 **exercises 2 days per week (20-40')**.

WARM-UP

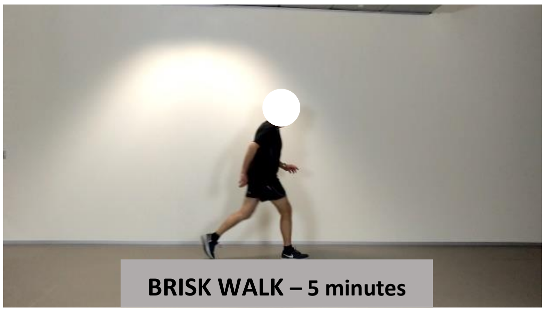

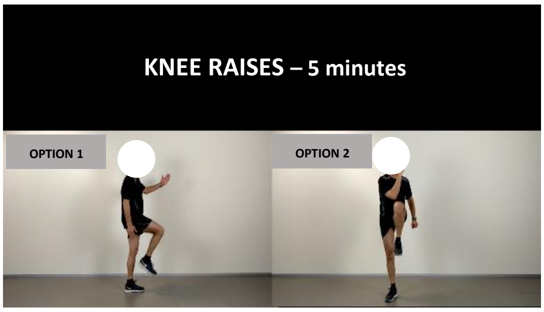

CIRCUIT OF EXERCISES

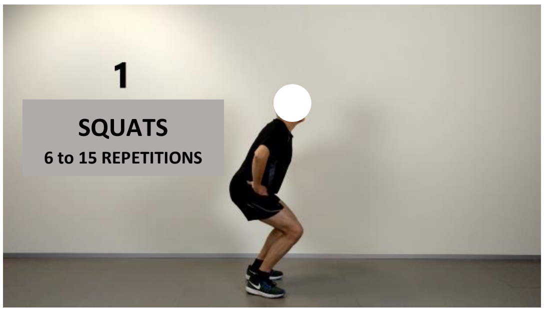

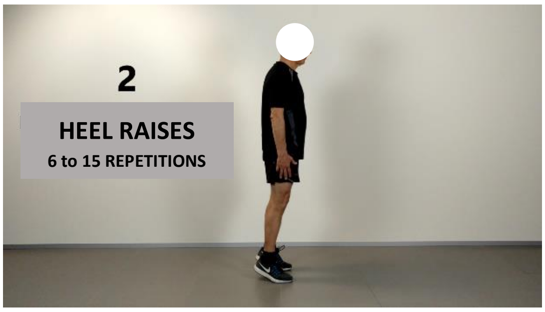

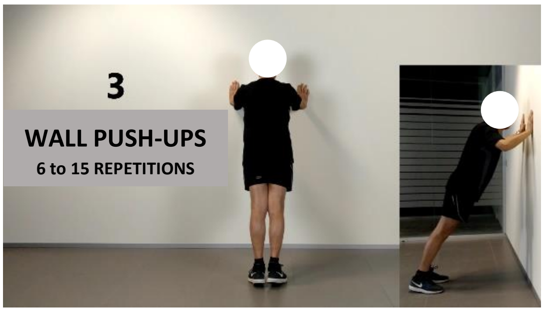

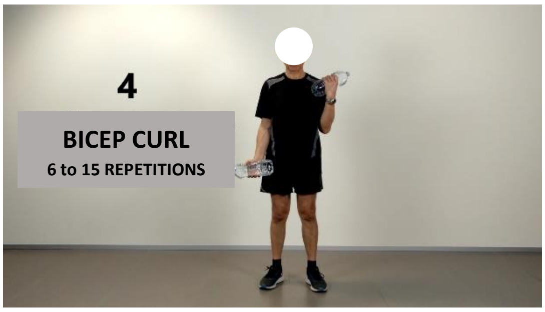

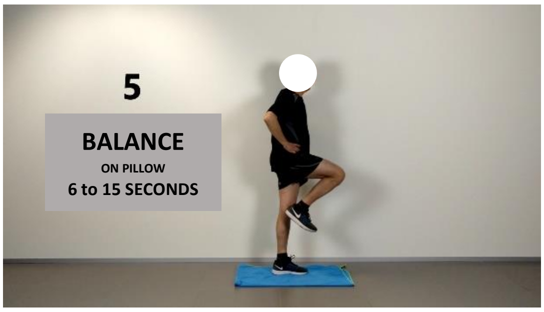

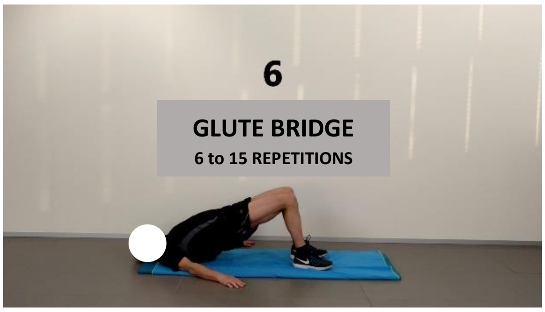

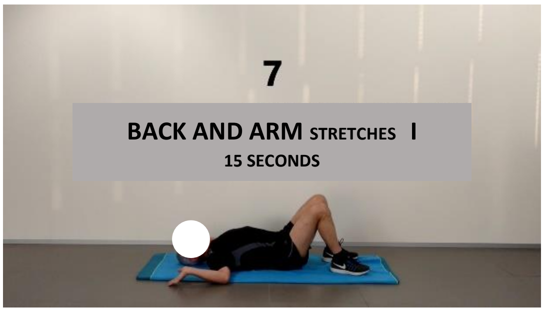

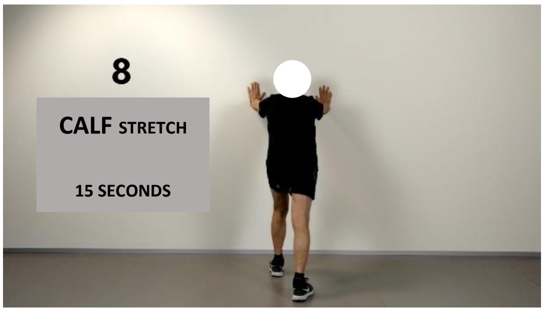

AEROBIC BRISK WALKING EXERCISE

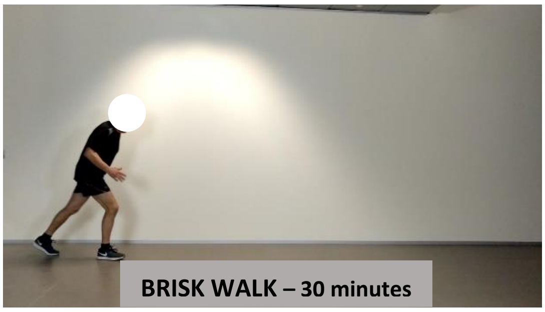

<u>**WEEKS 7 – 8:**</u> **Brisk walking 3 days per week (30')** and the following 12 **exercises 2 days per week (30-45')**.

WARM-UP

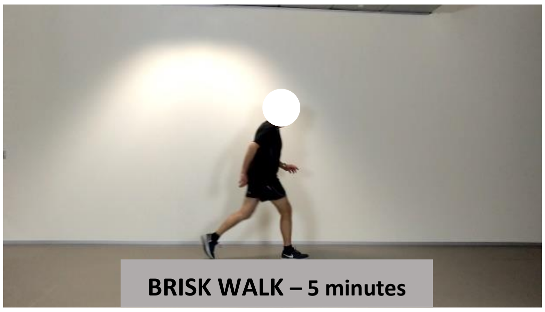

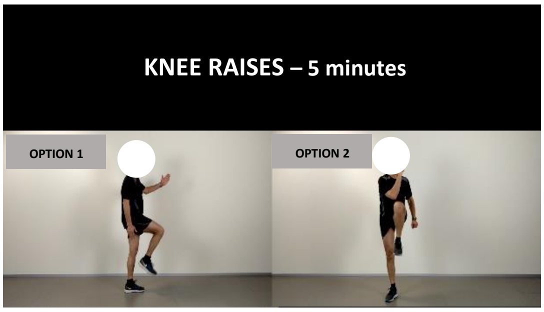

CIRCUIT OF EXERCISES

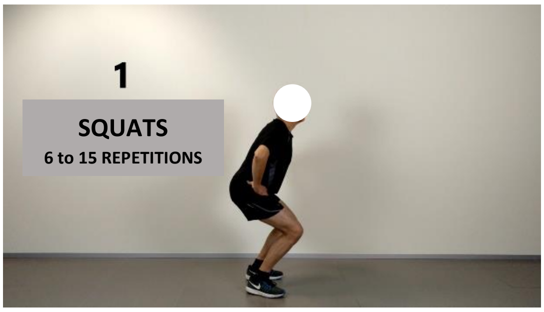

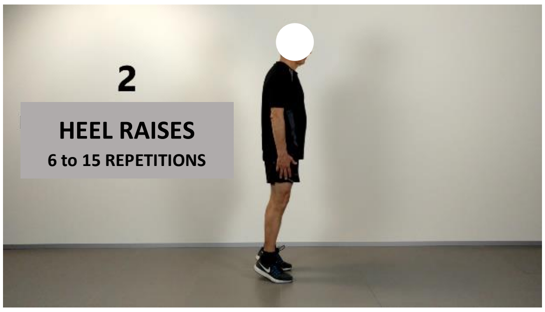

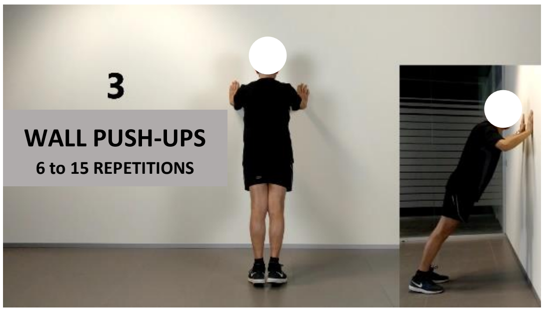

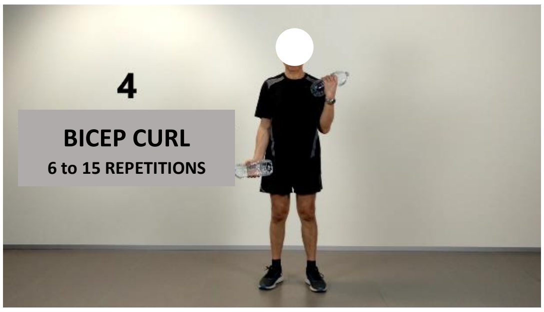

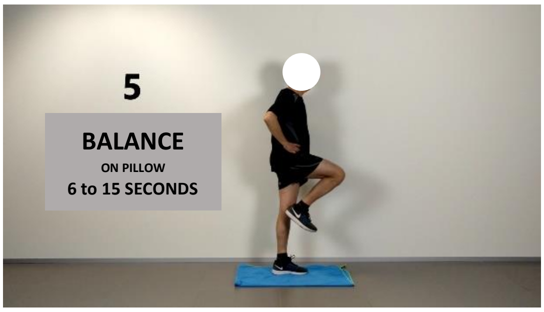

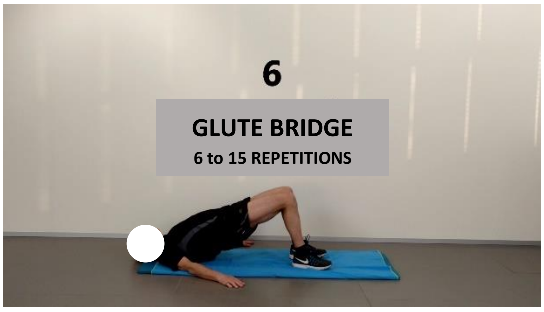

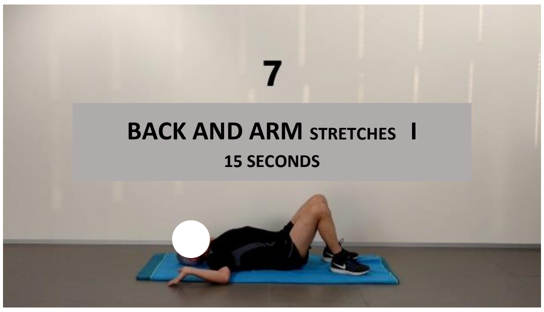

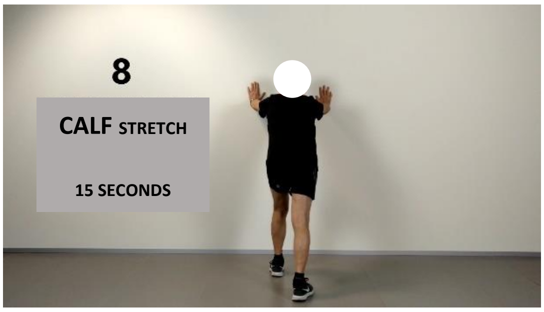

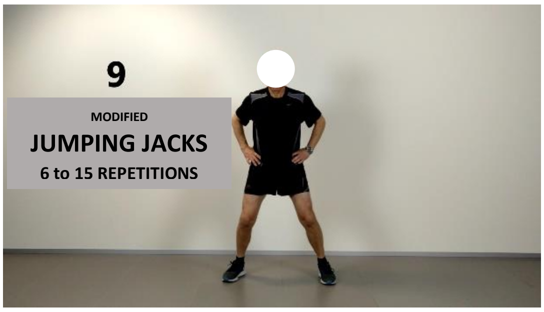

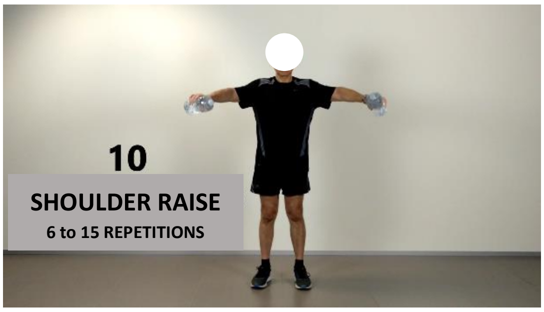

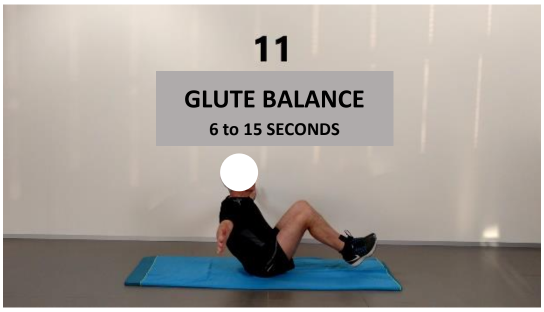

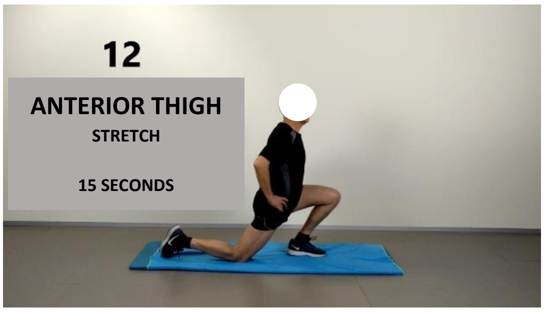

AEROBIC BRISK WALKING EXERCISE

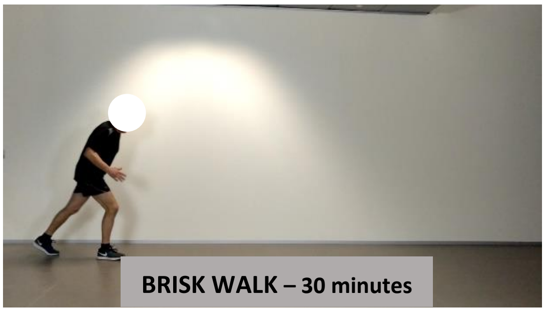

<u>**WEEKS 9 – 10:**</u> **Brisk walking 3 days per week (50')** and the following 12 **exercises 2 days per week (30-45')**.

WARM-UP

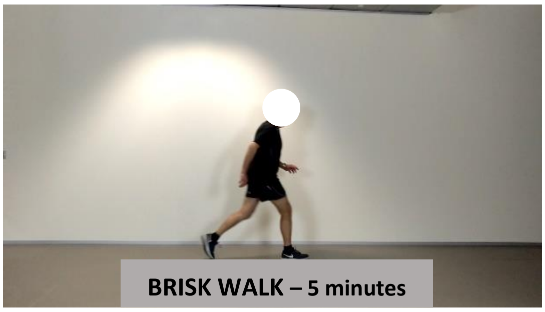

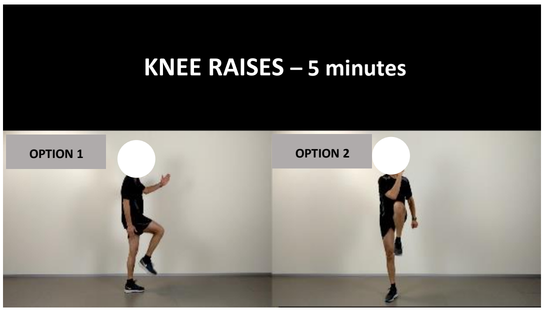

CIRCUIT OF EXERCISES

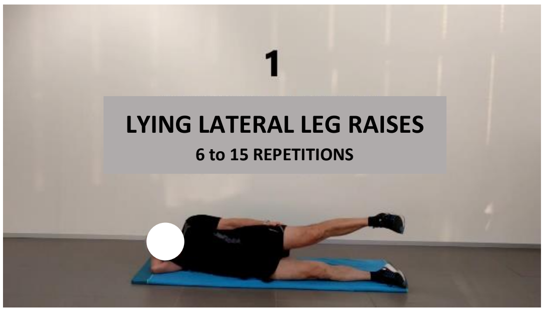

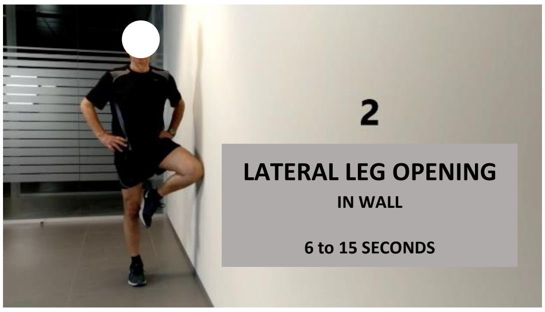

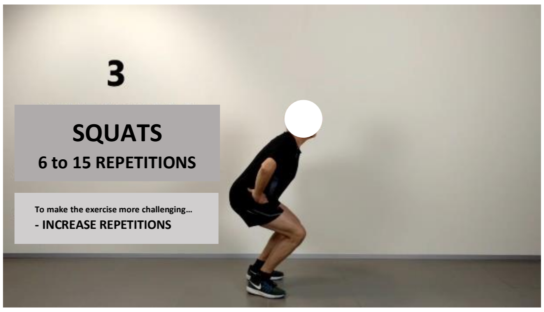

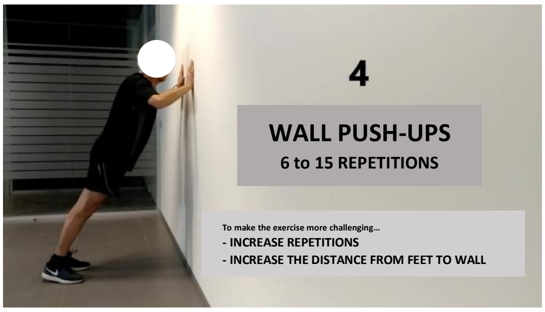

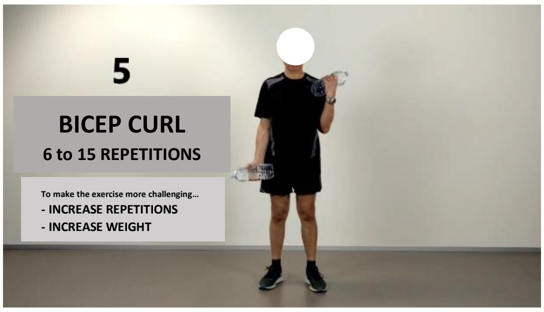

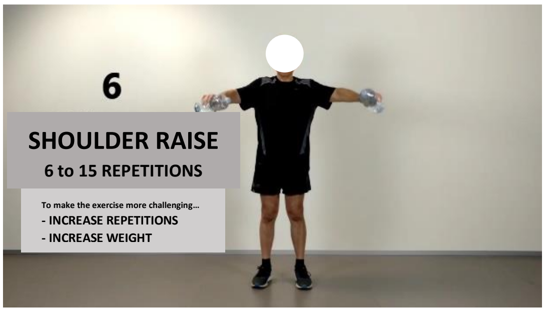

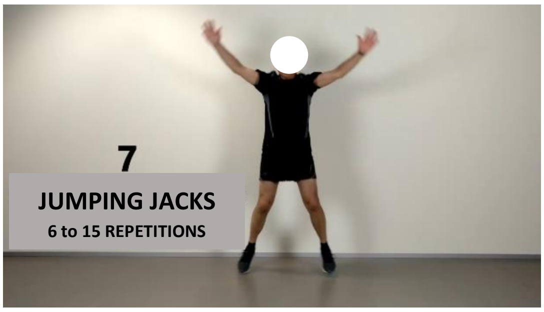

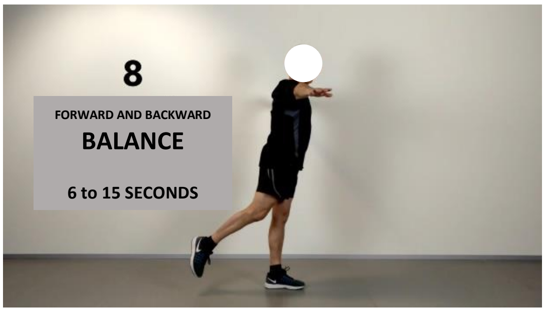

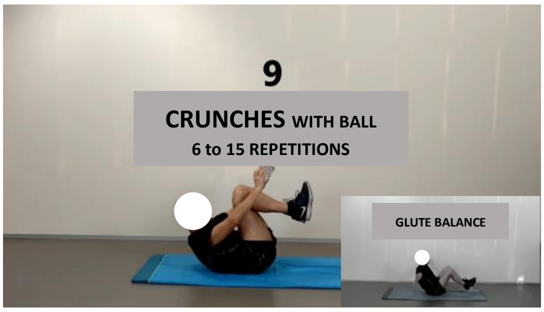

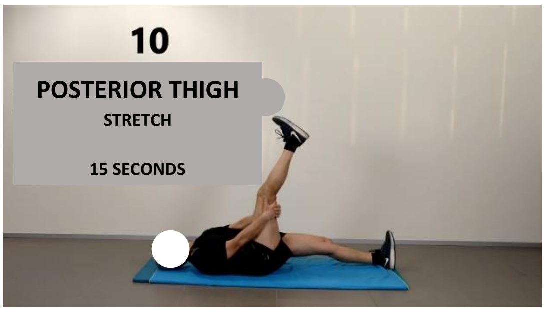

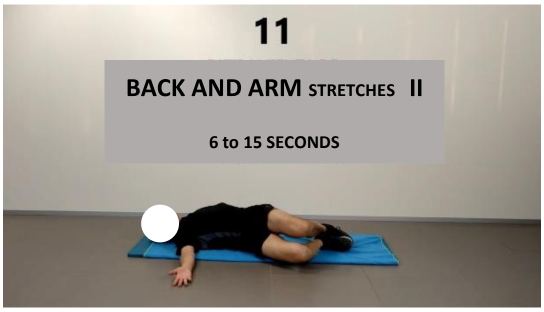

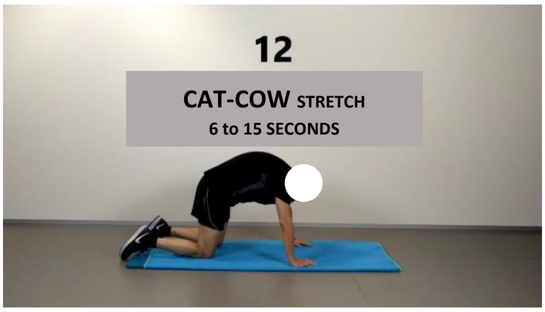

AEROBIC BRISK WALKING EXERCISE

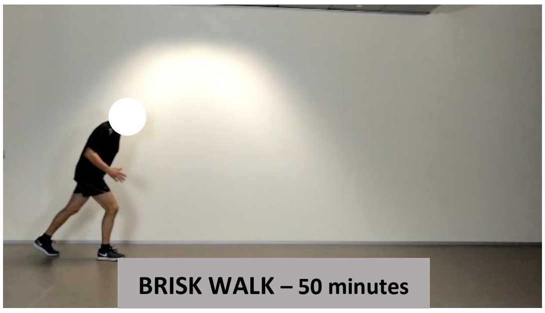

<u>WEEKS 11 – 12:</u> Brisk walking 4 days per week (50') and the following 12 **exercises 3 days per week (30-45')**.

WARM-UP

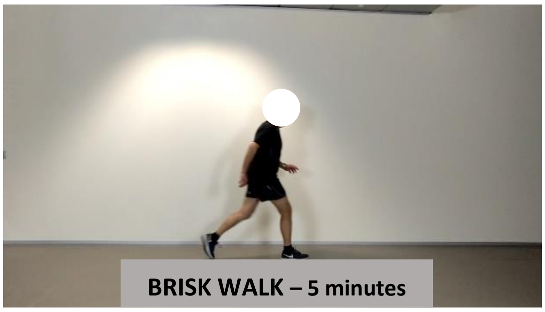

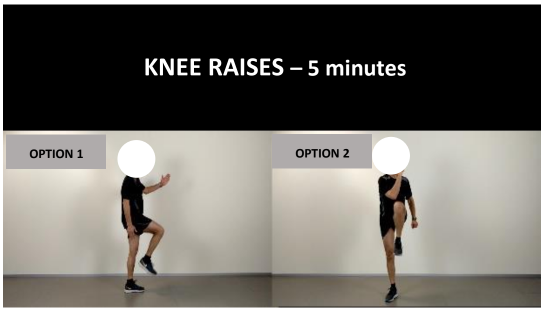

CIRCUIT OF EXERCISES

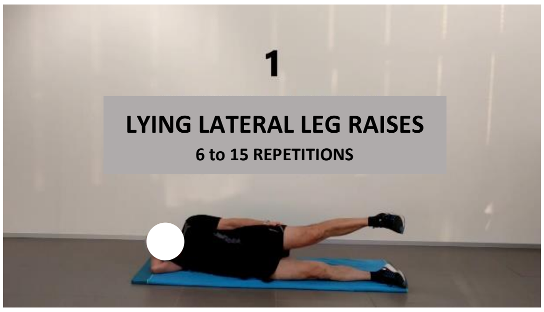

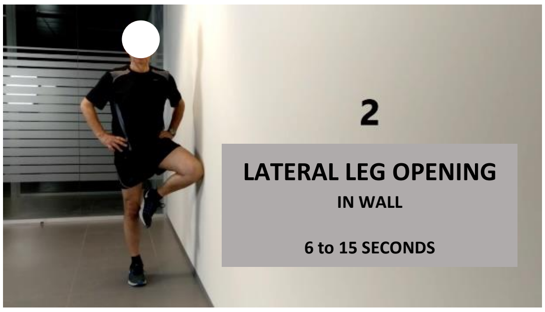

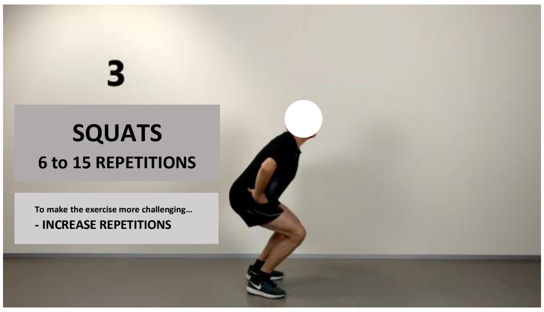

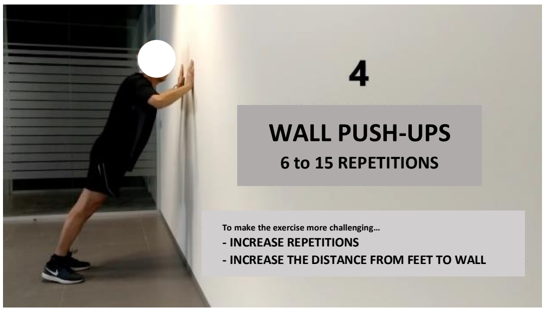

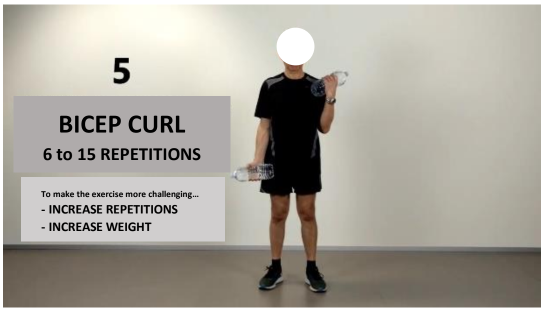

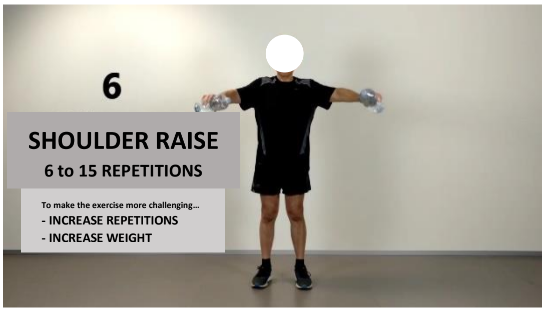

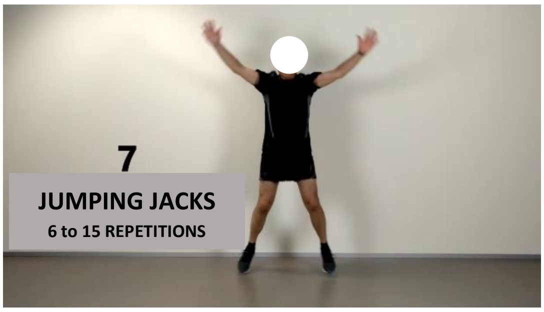

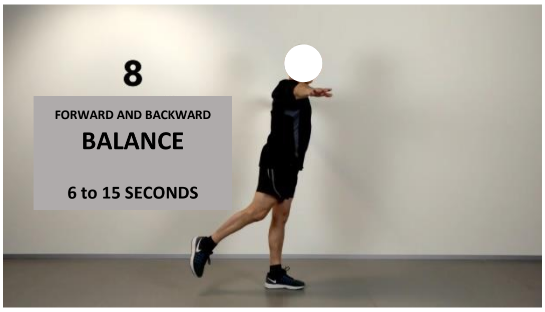

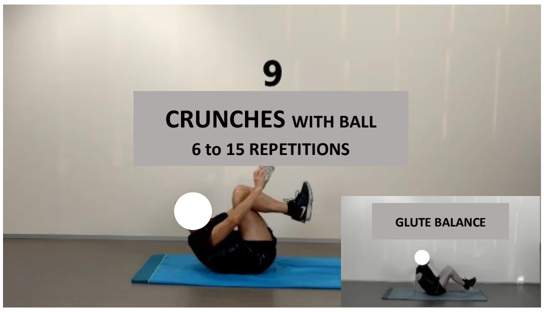

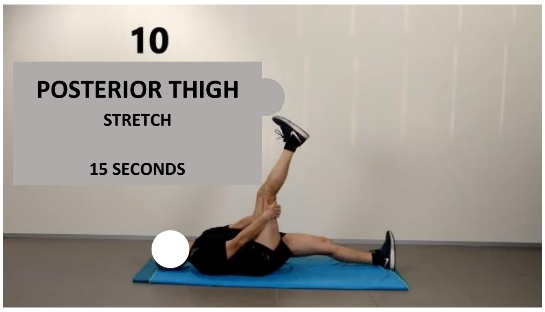

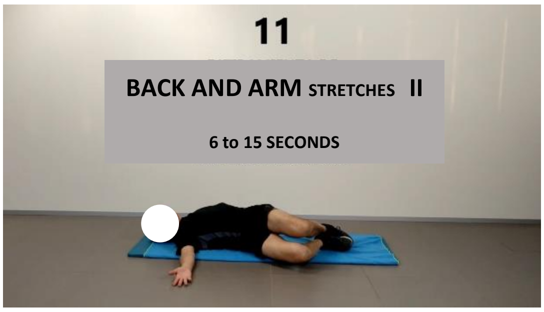

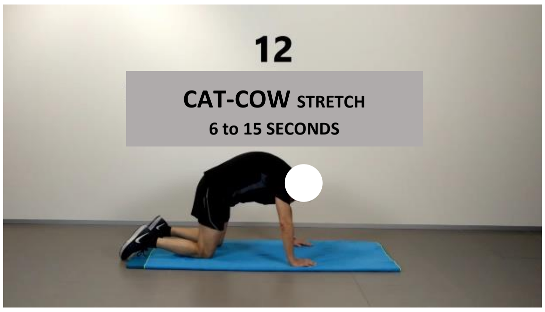

AEROBIC BRISK WALKING EXERCISE

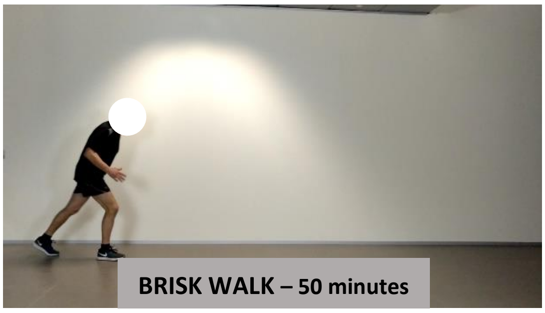

Supplement: Supplementary file 7 [file Data_Sheet_2.pdf]
